# Jobs and job quality between the eve of the Great Recession and the eve of COVID‐19

**DOI:** 10.1111/1475-5890.12279

**Published:** 2021-08-22

**Authors:** Pascale Bourquin, Tom Waters

**Affiliations:** ^1^ Institute for Fiscal Studies

**Keywords:** employment, jobs, recovery, job quality, Great Recession, COVID‐19

## Abstract

In 2019, the employment rate among 25‐ to 64‐year‐olds in the UK reached 80 per cent – the highest on record, and considerably higher than the 76 per cent rate recorded shortly before the Great Recession. In this paper, we investigate the growth in employment between the eve of the Great Recession and the eve of COVID‐19 across several dimensions. We analyse which sectors, demographic groups and regions accounted for the rise. We also investigate how job ‘quality’ – in both financial and non‐financial terms – has changed. We find that almost all demographic groups and regions saw a rise in employment, especially those with low pre‐existing employment rates and those near the bottom of the income distribution. Hourly pay growth was very weak over the period, with the median actually slightly falling. Other indicators of job quality show a more mixed picture: employees seem to have greater appreciation of their work and firm, but perceive less security and flexibility in their job.

## 1 INTRODUCTION

The 12 years between the eve of the Great Recession and the eve of the COVID‐19 pandemic saw substantial changes to the UK labour market. Employment fell in the wake of the recession before bouncing back strongly, leaving the employment rate in 2019 at record levels. Wage growth, however, was very weak, with median wages essentially unchanged. In this paper, we document in detail this chapter of the UK labour market.

Understanding the nature of the labour market over this time is important for at least three reasons. First, the recession and recovery were historically unusual. The rise in unemployment was relatively limited compared with previous recessions, but the effects on wages – and particularly the lack of strong wage growth in the aftermath of the recession – were comparatively large.^1^ It is therefore valuable to document precisely the shape of the labour market's fall and rise. Second, the labour market during this period sets the backdrop for the impact of COVID‐19 and the exposure of the UK economy to the shock that it induced. Third, as the economy returns to something more normal after the COVID‐19 crisis, the pre‐crisis labour market provides a benchmark for how much work there may be to do to recover ground that has been lost – as well as highlighting the areas, such as pay growth, where performance was lacking even prior to the pandemic.

This paper analyses which demographic groups, sectors and regions accounted for the rise in employment; the distribution of wage growth; and changes in job quality – all focusing mainly on those aged 25–64. While much has been written about the UK labour market since the Great Recession,^2^ the pandemic creates a defined end point of this episode, for which we provide a comprehensive history. We are also able to look at the variation in labour market experience along a wide range of dimensions, and examine the often neglected issue of job quality.

We find that overall employment grew very strongly between the two crises, led by women in full‐time work. The growth was concentrated at the bottom of the household income distribution, which implies an equalising effect on incomes. It was also widely shared: all major demographic groups (by gender, age, education, ethnicity, immigrant status, family type and region) saw an increase in employment, with the exception of individuals with degrees, who saw a very slight decrease.

While the picture for the number in employment is clearly very positive, it is more mixed for notions of ‘job quality’. The shares of the workforce looking for a different job, on a temporary contract and overemployed were each about the same in 2019 as they were in 2007. Underemployment rates remained somewhat elevated. Workers’ perceptions of and attitudes towards their jobs were also mixed. They were more likely to report that their job was interesting and valuable, that they had a good relationship with their firm and that they had good advancement opportunities. But workers were also more likely to report difficulties at work, including stress; there is also some evidence that they considered their job less secure and (surprisingly) less flexible.

The aspect of the labour market in this period that is clearly most negative is pay. Median hourly pay for 25‐ to 64‐year‐olds was, in real terms, slightly *lower* in 2019 than it was in 2007. Growth was somewhat stronger at the bottom of the distribution than further up, but by historical standards was still very weak.

The paper is organised as follows. We first describe the data that we use (Section 2). Next, we analyse which demographic groups, regions and industries saw the highest growth in employment (Section 3). We then quantify the extent to which the increase in employment was associated with changes in job quality, including pay (Section 4). Throughout, we mainly focus on those aged 25–64, as employment above these ages is relatively unusual (though becoming increasingly common) and because trends in employment below 25 are complicated by more people staying in education for longer.

## 2 DATA

We use four data sets in this paper, which we now describe in turn.

### 2.1 Labour Force Survey

The main data set that we use is the Labour Force Survey (LFS), which is a quarterly survey of the UK population conducted by the Office for National Statistics (2020). Participants are followed for five consecutive quarters in a rotating panel, though in this work we treat the data as a repeated cross‐section. The survey contains information on labour market outcomes, including industry, occupation, hours worked and earnings, as well as demographic information, such as immigration status, educational qualifications, and number of children. The sample size in 2019 was around 53,000 individuals per quarter. We use the weights supplied with the data which are calibrated to the UK population (based largely on Census data) using age, sex and location of residence.

The LFS is the source for the UK's official labour force statistics (employment, unemployment, number of self‐employed etc.). We do not use the earnings variables, which are subject to the usual concerns about measurement error in survey information on earnings and which have a significant amount of non‐response.^3^ Measures of hourly pay in the LFS also tend to give implausibly high numbers of people paid under the minimum wage, presumably indicating measurement error. Instead for earnings we rely on the Annual Survey of Hours and Earnings, described below. Response rates to the LFS have steadily declined over time, from 60 per cent in 2007 to 39 per cent in 2019,^4^ though Office for National Statistics (2018b) summarises research that uses LFS data linked to administrative data and shows that the bias resulting from non‐response is fairly minimal.

### 2.2 British Social Attitudes Survey

The British Social Attitudes Survey (BSAS) is an annual cross‐sectional survey of over 3,000 adults conducted by NatCen (2007 and 2017). It collects data on a wide variety of social, economic and political issues. Some questions are only asked to (random) subsets of the sample, and many questions are only asked in certain years of the survey. For our purposes, the main questions of interest are attitudes towards work and job satisfaction, which were asked in 2005 and 2015. In both years, these were only asked to a subset of the sample. The sample of workers who answered these questions was 422 in 2005 and 802 in 2015.

Many questions are asked by an interviewer, but the ones we are interested in are asked in a self‐completion questionnaire, possibly reducing interviewer influence. The questionnaire is either collected by the interviewer or can be sent later to NatCen. We use the weights supplied with the data which are calibrated to the population of Great Britain using age, sex and region. The response rate for the self‐completion questionnaire (conditional on being interviewed) was 85 per cent in 2015, and NatCen judges that no adjustment to the weights for self‐completion non‐response is required.^5^

### 2.3 Family Resources Survey

For household income analysis, we use the Family Resources Survey (FRS), an annual (fiscal year) repeated cross‐sectional survey of around 20,000 households conducted by the Department for Work and Pensions (2020). The data contain detailed information on incomes, including income from employee earnings, self‐employment, state benefits, savings, pensions and property. The data form the basis of the UK's official income statistics (for example, income inequality and poverty). We use the weights supplied with the data which are calibrated to the UK population using age, sex, region, number of children, housing tenure and council tax band. We use data covering the financial years 2007–08 and 2018–19. Response rates for the FRS have dropped from 59 per cent in 2010–11 to 50 per cent in 2018–19.^6^

### 2.4 Annual Survey of Hours and Earnings

The Annual Survey of Hours and Earnings (ASHE) is an employer survey of 1 per cent of employee jobs, with a sample of roughly 180,000, conducted by the Office for National Statistics (2019). The survey occurs in April each year. It contains information on earnings and hours worked, as well as some basic demographics about employees. It is used to produce the official statistics on the earnings distribution, and is widely considered the most reliable source for earnings data in the UK.^7^ Unlike in the LFS, only a very small share of jobs appear to be paid under the minimum wage. We use the weights supplied with the data which are calibrated to the UK population using occupation, region, age and sex.

## 3 THE RISE IN EMPLOYMENT

In this section, we describe the magnitude and nature of the growth in employment that was observed between 2007 and 2019. Section 3.1 presents longer‐term trends in different types of employment by sex for those aged 25–64 and Section 3.2 shows what this will have meant for poverty. Section 3.3 then investigates employment trends across demographic subgroups over the period, while Section 3.4 shows how the distribution of workers across industries has changed over time. Last, Section 3.5 explores trends in employment from 2007 to 2019 for individuals aged 16–24.

### 3.1 Trends in employment rates

**FIGURE 1:**
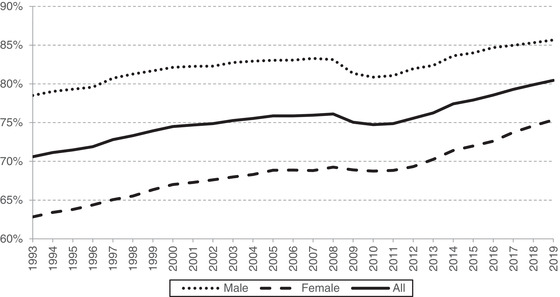
Employment rates by sex *Note*: Sample is individuals aged 25–64. *Source*: Authors’ calculations using Labour Force Survey, 1993 to 2019.

In 2019, around 28 million individuals aged 25–64 were in work, an employment rate of 80 per cent – the highest since records began (in 1971). Figure 1 shows trends since 1993. Both male and female employment were steadily increasing from the early 1990s up until the Great Recession. In the wake of the recession, male employment fell by about 2 percentage points (ppts) while female employment was fairly steady. From 2010, employment started to increase, leaving the rate 4.5ppts higher in 2019 than its 2007 level of 76 per cent.

Most of the post‐recession employment growth was driven by women. Female employment in 2019 stood 7ppts above its 2007 level, while male employment was just 2ppts above. This served to close the gender employment gap from 14ppts to 10ppts.

This can partly be explained by the huge change in working patterns at particular points in the life cycle observed for women over time, with far more women in their mid‐to‐late 20s and early 30s being in work in 2019. Women are having children both less frequently and later in life than they used to. When they do have children, they are also less likely to drop out of the labour market and tend to return to work faster.^8^ Two significant policy changes in the UK will have particularly affected women's labour supply – increases in work‐search requirements for lone parents with young children in 2008^9^ and the steady increase in the female state pension age between 2011 and 2020.^10^

**FIGURE 2:**
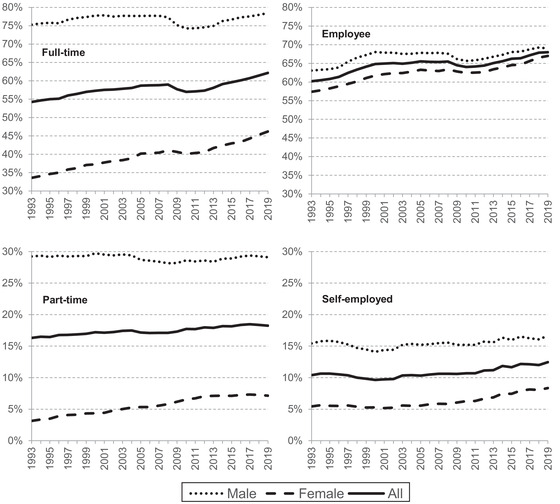
Part‐time, full‐time, self‐employed and employee rates, by sex *Note*: Sample is individuals aged 25–64. *Source*: Authors’ calculations using Labour Force Survey, 1993 to 2019.

Figure 2 demonstrates that much of the increase in the overall employment rate from 2007 to 2019 was driven by full‐time employment. This is particularly true for female full‐time work, which grew by 6ppts, continuing the steady rise seen since the early 1990s. In contrast, by 2019, full‐time male employment was only a little above its pre‐2007 level.

Roughly two‐fifths of the growth in employment between 2007 and 2019 was seen in self‐employment – a part of the workforce at which the government has found it especially difficult to precisely target insurance during the COVID crisis. While the share of the population working as employees dipped in the aftermath of the financial crisis, self‐employment rates continued to rise. Cribb and Xu (2020) show that the rise in self‐employment since 2007 was entirely driven by an increase in the ‘solo self‐employed’, who operate on their own without employees.

### 3.2 Employment and poverty

Given the size of the increase in employment from 2007 to 2019, it is natural to ask what impact this had on household incomes. In this subsection, we explore where in the household income distribution the employment growth occurred, in order to gauge what employment growth has meant for the living standards of those who are disproportionately on low incomes.

**FIGURE 3:**
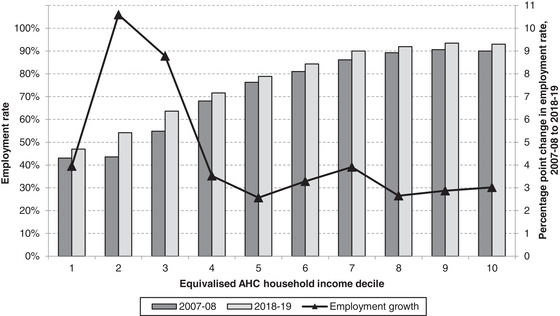
Change in employment by household income decile, 2007–08 to 2018–19 *Note*: Incomes have been measured net of taxes and benefits and after housing costs (AHC) have been deducted. All incomes have been equivalised using the modified OECD equivalence scale. Sample is individuals aged 25–64, though income deciles are calculated based on the whole population. *Source*: Authors’ calculations using Family Resources Survey, 2007–08 and 2018–19, and a ‘top incomes’ adjustment using administrative tax data.

Figure 3 shows employment growth from 2007 to 2019 by household income decile. To be clear, here we are using repeated cross‐sectional data, and so we are simply comparing the employment rates of those who are in a given decile in 2007 and those who are in that same decile in 2019, who need not be the same people. The sharpest growth in employment (of 25‐ to 64‐year‐olds) was seen in the second and third household income deciles, with 11ppt and 9ppt increases in their employment rates respectively. Employment in the remaining deciles increased by around 3–4ppts.

That these patterns had an equalising effect on employment rates across the distribution will have implications for poverty. Though it is difficult to precisely estimate by how much employment growth will have kept poverty down, it was clearly a key driver of income growth among low‐income households. It is therefore likely to have been a significant factor in keeping poverty lower than it otherwise would have been. The current crisis resulting from the COVID‐19 pandemic could mean that much of this may be undone, likely leaving many low‐income households vulnerable. Looking at the types of people who were brought into work from 2007 to 2019 can therefore help provide a picture of the pre‐pandemic circumstances of those below and around the poverty line.

### 3.3 Change in employment rates for different groups

We now turn to exploring in more detail where the growth in employment from 2007 to 2019 occurred.

**FIGURE 4:**
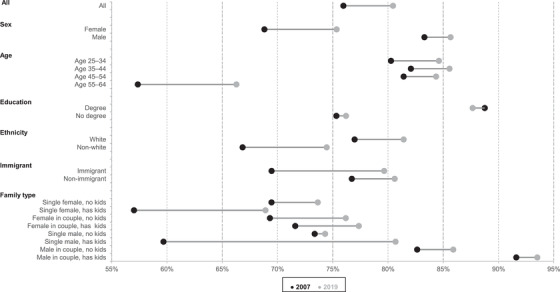
Change in employment rates by various characteristics, 2007 to 2019 *Note*: ‘Immigrants’ are defined as those who first lived in the UK aged 16 or older. Sample is individuals aged 25–64. *Source*: Authors’ calculations using Labour Force Survey, 2007 and 2019.

Overall, the largest increase in employment was experienced by population subgroups that historically had lower employment rates.^11^ This can be observed in Figure  4, which presents the changes in the employment rates for different demographic groups from 2007 to 2019.^12^

Notably, employment rates increased for all of these groups^13^ with the exception of individuals with a degree, for whom the employment rate decreased by 1ppt. This is likely at least in part due to the huge rise (4.7 million) in the number of 25‐ to 64‐year‐olds with a degree between 2007 and 2019. This means that degree holders are simply a different kind of group, on average, from the group they were in the past, which probably makes accurate like‐for‐like comparisons over time impossible (the same is likely true for those without a degree).

Three groups saw particularly large increases in employment. First, single mothers’ employment increased by 12ppts, a rise partly caused by policy reforms incentivising paid work (as mentioned above).^14^ Most of this rise was in part‐time employment (see Figures A1 and A2 in the online appendix). It is worth noting that employment amongst lone parents, a central part of the Labour governments’ child poverty strategy, had already increased from 47 per cent to 57 per cent in the decade leading up to the Great Recession.

Second, the employment rate among those aged 55–64 increased by 9ppts. Again, a particular policy reform – the rising female state pension age – played a key role. Given this, it is not surprising that the increase in employment among older workers was stronger among women (+13ppts) than among men (+5ppts).

Third, immigrants saw a 10ppt rise in employment. Together with an increase in the number of immigrants in the UK, this led to around 2 million more immigrants in work. The increase in the employment rate for immigrants can partly be explained by a change in their composition. There has been a clear shift from low‐educated to highly educated immigrants and a tilt towards those from the (mainly eastern European) countries in Europe that were not in the EU prior to 2004, and away from Africa, the Americas and Oceania. Both of these effects represent a movement towards immigrant groups that are more likely to be employed. The change in composition with regards to country of birth may have been driven in part by the loosening of restrictions on immigration from eastern Europe and tighter controls on immigration from outside the European Economic Area seen over the period.^15^

The employment rate for non‐immigrants also increased between 2007 and 2019, albeit to a lesser extent (4ppts). A common question in the policy debate is whether the substantial increase in the number of immigrants in the UK affected the employment of non‐immigrants. While it is possible that the increase in the employment rate for non‐immigrants would have been higher in the absence of immigrants, in general the empirical evidence suggests very little employment effects of that kind.^16^

The increases in employment have also meant a decline in household worklessness. As well as the employment increases among single households visible in Figure 4, Appendix Table A3 shows that there was also a fall in the share of coupled households where no one was in work.

**FIGURE 5:**
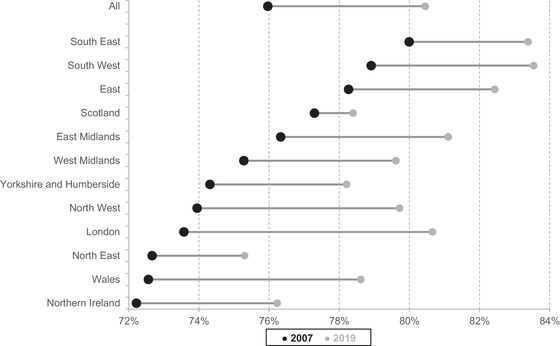
Change in employment rates by region, 2007 to 2019 *Note*: Sample is individuals aged 25–64. *Source*: Authors’ calculations using Labour Force Survey, 2007 and 2019.

Figure 5 presents changes in the employment rate between 2007 and 2019 by region. Every region shared in the employment growth over this period, though with considerable variation. On average, the lower‐employment parts of the UK saw faster growth (with the exception of Northern Ireland and the North East).

The change in employment rates of non‐immigrants by region was similar to that of the total population, with the exception of London, where the employment growth for non‐immigrants was considerably (3ppts) lower. We do not find substantial differences in employment growth across regions by sex: for every region, female employment growth was around 2–7ppts higher than that of males (overall female employment growth was 4ppts higher).

The preceding evidence has shown the largest growth in employment among women, immigrants and Londoners. One might ask whether the especially strong growth among these groups masked reduced employment among others. Evidence for this is hard to find, however. For example, even for non‐immigrant men living outside of London, the overall employment rate increased by 1ppt from 2007 to 2019, though this was driven by an increase in part‐time employment. Full‐time employment in 2019 for this group was at the same level as in 2007.

### 3.4 Change in distribution of workers across industries

So far, we have investigated the sorts of people who saw increases in employment over the period; we now turn to the industries they worked in. Table 1 shows the change in the number of workers in each industry in absolute terms and as a share of the total workforce.

**TABLE 1:** Change in number and share of workers by industry, 2007 to 2019

	Number of workers (million)			As share of workforce		
	**2007**	**2019**	**Difference**	**2007**	**2019**	**Difference (ppts)**
Wholesale, retail, transportation	4.4	4.5	2%	18%	16%	–1.7
Manufacturing	3.1	2.6	–15%	13%	9%	–3.2
Human health and social health activities	3.1	3.9	26%	13%	14%	1.4
Professional activities incl. finance	2.5	3.7	48%	10%	13%	3.2
Education	2.5	3.0	24%	10%	11%	0.9
Construction	2.1	2.0	–2%	8%	7%	–1.2
Public admin and defence, social security	1.9	1.9	3%	8%	7%	–0.7
Administrative and support services	1.3	1.3	–4%	6%	5%	–0.8
Accommodation and food services	0.8	1.1	38%	3%	4%	0.7
Miscellaneous	1.0	1.5	51%	4%	5%	1.4
Information and communication	1.0	1.3	20%	4%	5%	0.3
Agriculture, forestry & fishing, mining & quarrying	0.4	0.4	–7%	2%	1%	–0.3
Electricity, gas, water supply, waste management etc.	0.3	0.4	23%	1%	1%	0.1

*Note*: ‘Miscellaneous’ includes arts, entertainment and recreation as well as other service activities, activities of households as employers and activities of extraterritorial organisations and bodies. Sample is individuals aged 25–64.

*Source*: Authors’ calculations using Labour Force Survey, 2007 and 2019.

Two public‐sector‐dominated industries – health and education – both saw significant increases over the period. An ageing population is likely to have boosted work in the former, with the strongest increases among those working in residential and social care. The rise in the number of people working in education (0.5 million) appears to have been driven by those working in the private sector or universities, with the increase there considerably greater than the rise in the number employed in public sector education (reported to be 0.1 million by Cribb, Davenport and Zaranko (2020)).

There were also significant increases in the number of workers in the hospitality sector, in particular catering, as well as in professional activities, with more people working in management consultancies, head offices, engineering and architecture. There were falls in the share of workers working in wholesale, retail and transportation, and in manufacturing – with the latter being the continuation of a long‐run decline in the sector.

Overall, these trends show increases in typically lower‐paying industries (such as accommodation and food services or human health) as well as higher‐paying ones (such as professional services). Section 4 looks at this issue further, investigating whether the increase in the employment rate from 2007 to 2019 was accompanied by a change in the pay and quality of jobs.

### 3.5 Younger workers

Thus far, our focus has been on workers aged 25–64. We now briefly investigate employment patterns among younger workers (16–24). While almost all demographic groups saw an increase in employment from 2007 to 2019, those aged 16–24 saw a 3ppt decline. We examine this decline further in Table 2, which lists the economic activity of 16‐ to 24‐year‐olds in 2007 and 2019. There are two things to note from the table. First, because unemployment fell across the period, the decline in labour force participation (employment or unemployment) was considerably larger than the fall in employment alone. Second, this decline was entirely accounted for by an increase in the share of 16‐ to 24‐year‐olds in full‐time education (likely driven in part by the raising of the school (or training) leaving age). Among those *not* in full‐time education, the 16–24 employment rate increased by 2ppts.

**TABLE 2:** Economic activity of those aged 16–24, 2007 and 2019

	2007	2019	Change (ppts)
Employee	55.6%	52.1%	–3.5
Self‐employed	2.3%	2.7%	0.4
Unemployed	10.2%	7.6%	–2.6
Long‐term sick	1.4%	2.1%	0.6
In full‐time education	23.2%	29.7%	6.5
Looking after family	4.2%	2.3%	–1.8
Other inactive	3.1%	3.5%	0.4

*Source*: Authors’ calculations using Labour Force Survey, 2007 and 2019.

## 4 HOW DID THE QUALITY OF JOBS CHANGE BETWEEN THE GREAT RECESSION AND THE COVID‐19 CRISIS?

Thus far, we have described the rise in employment between 2007 and 2019. But the relationship between families’ living standards and paid work is dependent upon not just the number of workers, but also the nature of the jobs they do. In this section, we investigate how job quality changed over the period. By ‘job quality’ we mean the value that workers might get out of the job they have, rather than a broader notion such as how well the job contributes to societal welfare.

**FIGURE 6:**
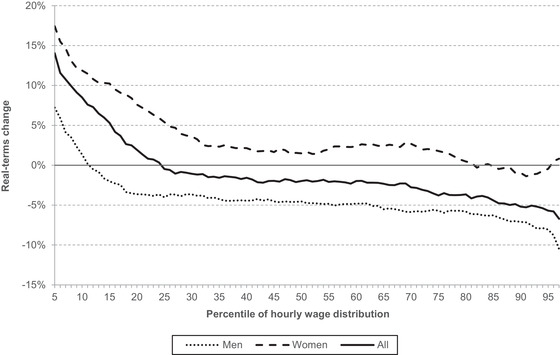
Change in real hourly employee earnings, 2007 to 2019 *Note*: Wages are deflated using CPIH. Percentiles 1–4 and 98 and 99 are excluded because of high statistical uncertainty. Sample is individuals aged 25–64. *Source*: Authors’ calculations using the Annual Survey of Hours and Earnings, 2007 and 2019.

The most straightforward indicator of job quality is hourly pay, since it measures the financial reward a worker receives for an hour of their time. Figure 6 shows changes in real hourly earnings among employees (aged 25–64) across the wage distribution between 2007 and 2019, split by sex. This figure treats the data as a repeated cross‐section, comparing the level of wages at a given percentile in 2007 and the level of wages at that same percentile in 2019.

Women saw faster growth than men, with female hourly pay rising almost across the board and all but the bottom 10 per cent of male wages actually falling in real terms. Wage growth has also been a little stronger at the middle of the distribution than the top, and much stronger at the bottom than the middle – a consequence partly of rises in the minimum wage (as discussed in Cribb, Norris Keiller and Waters (2018)). (Note that if one examines household total earnings, rather than individual hourly pay, the opposite trend emerges, with growth weaker further down the distribution than further up.)

However, by historical standards, the decade or so following the recession was a very bad one indeed for pay growth. At the median, overall real hourly pay fell by about 2 per cent; even at the 10^th^ percentile, it only grew by 9 per cent. In comparison, in the decade before the recession, median hourly earnings grew by 24 per cent.^17^ In other words, whereas we would usually expect job quality as measured by wages to improve over time, between 2007 and 2019 there was, for most jobs, no improvement at all – and even among lower‐paid jobs the improvement was fairly meagre.

One might worry that the trends here are misleading because of changes in other forms of compensation over the period (an important factor in US earnings inequality^18^). By far the most important form of non‐wage compensation in the UK is employer pension contributions; and this is a particularly important issue in this period because, starting in 2012, the government introduced a scheme of automatic enrolment into workplace pensions.^19^ By April 2019, this required a minimum of a 3 per cent contribution from employers on ‘qualifying earnings’ (which amounts to strictly less than 3 per cent of total earnings). Data limitations^20^ prevent us analysing the effect of this policy fully. But it is unlikely to make much of a difference: public sector workers (roughly a fifth of the workforce) are largely unaffected by the policy, and among private sector workers it increased workplace pension enrolment by about 37ppts.^21^ Combined with a contribution rate of less than 3 per cent, a back‐of‐the‐envelope calculation suggests that the effect on average total compensation would be under 1 per cent.

**FIGURE 7:**
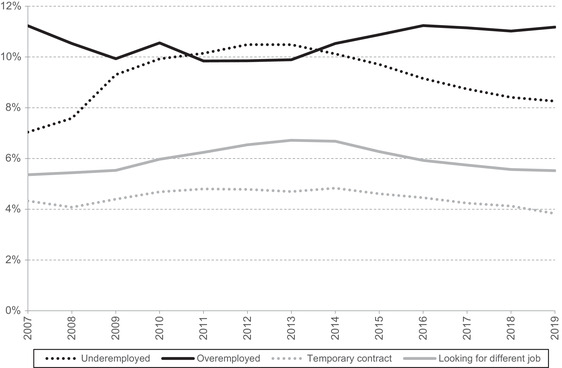
Job quality indicators *Note*: Lines show the percentages of workers aged 25–64. ‘Underemployment’ is comprised of respondents who are looking for another job and would like more hours in that job, plus those who are not looking for another job and would like more hours in their current job. ‘Overemployment’ is comprised of respondents who are looking for another job and would like fewer hours in return for less total pay in that job, plus those who are not looking for another job and would like fewer hours at their current job in return for less pay. *Source*: Authors’ calculations using Labour Force Survey, 2007–19.

Going beyond purely financial measures, Figure 7 analyses changes in job attributes and whether workers are looking for another job. Two measures relate to hours: underemployment and overemployment rates, where the worker would rather work more or fewer hours than he or she actually does. The figure also shows the share of workers who are on a temporary contract and the share who are looking for a different job. One measure that the figure does not include – and which is frequently the subject of political and media attention – is zero‐hours contracts (where the employee is not guaranteed any hours). We exclude this indicator because of measurement difficulties. The number of workers reporting being on a zero‐hours contract in the Labour Force Survey increased dramatically between 2012 and 2016 (especially 2012 to 2013). While that may in part reflect a genuine increase in their frequency, it is also likely partly driven by a greater awareness of zero‐hours contracts.^22^

With the exception of overemployment, all of the measures in the figure tell a similar story. Following the onset of the recession and the decline in employment, these indicators worsened. Even after employment started to recover (from 2010), these indicators continued to worsen until 2012–14, perhaps as unemployed workers getting back into work accepted jobs they would in other times have turned down. These indicators began to reverse course in 2013–14, and by 2019 they were roughly back to their pre‐recession levels, though underemployment remained slightly elevated. The rate of overemployment shows the mirror image: it became slightly less frequent following the recession, and then increased back to its pre‐recession level. Changes since the recession in these indicators are very similar for men and women, although the levels of overemployment, underemployment and temporary status are somewhat higher for women (the share looking for a different job is about the same). If we restrict our attention to non‐immigrant men outside of London – a group that one might think would have potentially done less well out of the job growth – the patterns are again similar. Trends are also similar if we look just at those in the bottom quarter of the earnings distribution, though the levels of underemployment are much higher and overemployment much lower.

**FIGURE 8:**
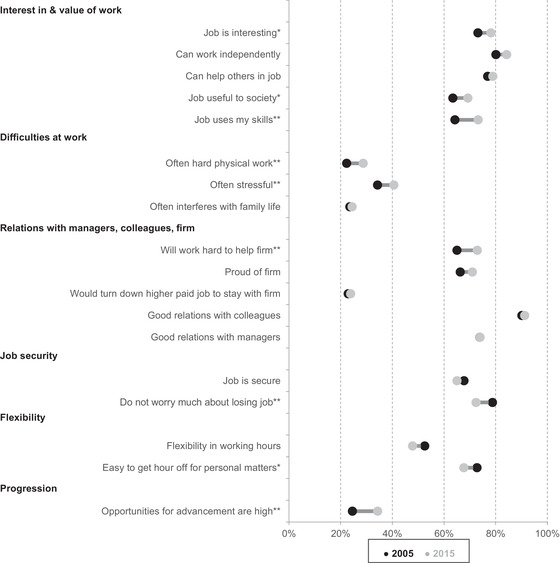
Attitudes to and perceptions of job, 2005 and 2015 (workers aged 25–64) *Note*: * indicates a statistically significant difference at the 10 per cent level; ** indicates a statistically significant difference at the 5 per cent level. *Source*: Authors’ calculations using British Social Attitudes Survey, 2005 and 2015.

Another way of measuring job quality is analysing workers’ attitudes to and perceptions of their job. Figure 8 displays a variety of job quality indicators using data from the British Social Attitudes Survey. The questions are only asked of BSAS respondents in 2005 and 2015 – a slightly different period from the one that we analyse in the rest of this paper. Sample sizes in BSAS are relatively small, and so we indicate statistically significant differences with asterisks.

The figure shows several dimensions along which job qualities have improved, and several along which they have worsened. Workers were more likely to consider their job interesting and valuable in 2015 than they were in 2005. There is some evidence that their relationship with the firm they work for improved. And the fraction reporting that ‘opportunities for advancement are high’ in their job increased from 25 per cent to 34 per cent. However, workers were more likely to report difficulties at work, including stress and (perhaps surprisingly) hard physical work. There is also some evidence that in 2015 they considered their job less secure than they did in 2005. Though it might be thought that greater flexibility can be the flipside of less security (for example, because some gig economy workers have reduced employment rights but more control over their hours of work from one week to the next), in fact perceptions of flexibility also appear to have, if anything, worsened on average over the period.

Investigating differences in these trends across different subgroups is somewhat hampered by BSAS's small sample sizes. We adopt a simple procedure: we average values within each of the categories in Figure 8 (for example, ‘interest in & value of work’), and compare changes over time between men and women, and between workers above and below median earnings. The only statistically significant difference is in ‘difficulties at work’, where lower‐paid workers saw significantly larger increases than higher‐paid ones (who saw very little change at all). Given the multiple hypothesis testing involved in this procedure and the modest significance of the difference (*p* = 0.03), this should probably be taken with a grain of salt.

Taken together, we see a mixed picture for changes in job quality between the Great Recession and the current crisis. By the end of the period, workers appeared to be more interested in their work, have a better view of the firm they work for and perceive better opportunities for advancement. There was relatively little change in dissatisfaction with hours worked, and in the frequency of people on a temporary contract or looking for a different job. But on some dimensions, job quality declined: workers reported greater difficulties at work such as stress, less flexibility and less security. Moreover, hourly pay – the aspect of job quality that we would usually expect to steadily improve over time – fell across three‐quarters of the distribution, a very poor showing by historical standards.

## 5 CONCLUSION

The two key characteristics of the labour market over the period bookended by the Great Recession and the onset of the COVID‐19 crisis were the strong employment growth and the weak pay growth. The former was widely shared, and was strongest for those demographic groups that started out with low employment rates – including immigrants, lone parents and older workers.

The weak pay growth probably stands out as the worst attribute of the labour market over the period: at the median, hourly pay actually fell slightly, and though wages grew faster at the bottom of the distribution, the pace was fairly meagre by historical standards. Other characteristics of job quality give a more mixed picture. While employees seem to have greater attachment to their work and their firm, they also perceive less security and flexibility in their job.

In terms of living standards and poverty, there were certainly plenty of challenges before the COVID‐19 crisis – the weakness in earnings growth and benefit cuts had been putting a lot of pressure on incomes at the bottom. But there is no question that large falls in unemployment, and particularly in household worklessness, had been a significant factor in keeping poverty lower than it would otherwise have been. The current crisis may mean that much of that will be undone, with few countervailing forces to prevent more vulnerable households from falling into hardship. A key question for policymakers, with hugely important implications for living standards, is what can be done to ensure that employment will bounce back quickly when the temporary support is unwound.

## Supporting information

TABLE A1. Number of people in work by various characteristics, 2007 and 2019
2
TABLE A2. UK population by various characteristics, 2007 and 2019
TABLE A3. Change in employment rates amongst couples, 2007 to 2019
FIGURE A1. Change in part‐time employment by various characteristics, 2007 to 2019
|FIGURE A2. Change in full‐time employment by various characteristics, 2007 to 2019

## Footnotes

1 Cribb, Hood and Joyce, 2017.

2 For example, Blundell, Crawford and Jin (2014), Blanchflower (2015) and Clarke and Cominetti (2019).

3 See Britton, Shephard and Vignoles (2015) for a comparison of LFS earnings information with that in administrative data.

4
https://www.ons.gov.uk/employmentandlabourmarket/peopleinwork/employmentandemployeetypes/methodologies/labourforcesurveyperformanceandqualitymonitoringreports.

5
https://bsa.natcen.ac.uk/latest‐report/british‐social‐attitudes‐33/technical‐details.aspx.

6
https://www.gov.uk/government/collections/family-resources-survey--2. We have been unable to locate response rates for years before 2010–11.

7 See, for example, Gregg, Machin and Fernández‐Salgado (2014).

8 Roantree and Vira, 2018.

9 Avram, Brewer and Salvatori, 2018.

10 Cribb, Emmerson and Tetlow, 2016.

11 Note that we do not present the employment rate for people with and without disabilities, given that there have been multiple changes in the survey questionnaire with regards to the definition of being disabled and we thus are not able to construct a consistent measure of disability over time (see figure 5.1 of Cribb, Norris Keiller and Waters (2018)).

12 The extent to which changes in the employment rate by specific characteristics will have affected the overall employment rate will also depend on the number of people in each subgroup who were initially in work and the relative sizes of these groups. Table A1 in the online appendix shows the change in the number of people in each subgroup who are in work from 2007 to 2019, while Table A2 presents the change in the number of people in each subgroup as a share of the total population.

13 Excluding immigrants from the results presented does not change results substantially.

14 Single fathers saw an even larger rise, but are a very small group.

15 A fuller discussion of changes in immigration over the period is available in the working paper version of this article (Bourquin and Waters, 2020).

16 Dustmann, Fabbri and Preston, 2005; Wadsworth, 2018.

17 This statistic relates to all employees, rather than those aged 25–64.

18 See, for example, Pierce (2010).

19 See Cribb and Emmerson (2016) for a description.

20 The variant of ASHE we have access to does not have information on employer contributions beyond 2016. The trends between 2007 and 2016 are very little changed if we include employer pension contributions.

21 Cribb and Emmerson, 2016.

22 This point, and a comparison using business surveys of zero‐hours contracts, is discussed in greater detail in Office for National Statistics (2018a).
