# Supplementary material for: Jobs and job quality between the eve of the Great Recession and the eve of COVID‐19
Source: Fisc Stud. 2021 Aug 22;43(1):63–78. doi: 10.1111/1475-5890.12279 (PMC8441802; doi:10.1111/1475-5890.12279)
Supplement: Supplementary file 1 — TABLE A1. Number of people in work by various characteristics, 2007 and 2019 2 TABLE A2. UK population by various characteristics, 2007 and 2019 TABLE A3. Change in employment rates amongst couples, 2007 to 2019 FIGURE A1. Change in part‐time employment by various characteristics, 2007 to 2019 |FIGURE A2. Change in full‐time employment by various characteristics, 2007 to 2019 [file FISC-43-63-s001.pdf]

Appendix

TABLE A1. Number of people in work by various characteristics, 2007 and 2019

	Number in work (million), 2007	Number in work (million), 2019	Difference
**All**	24.6	27.7	12.6%
**Female**	11.3	13.1	16.2%
**Male**	13.3	14.6	9.5%
**Aged 25–34**	6.4	7.6	18.3%
**Aged 35–44**	7.6	7.2	–5.4%
**Aged 45–54**	6.5	7.6	16.7%
**Aged 55–64**	4.1	5.4	30.1%
**Degree**	6.3	11.0	75.2%
**No degree**	17.6	16.2	–7.6%
**White**	22.5	24.2	7.8%
**Non-white**	2.1	3.5	62.1%
**Immigrant**	2.4	4.4	84.9%
**Non-immigrant**	22.2	23.3	4.9%
**Single female, no kids**	2.2	2.7	22.4%
**Single female, has kids**	1.0	1.1	17.5%
**Female in couple, no kids**	4.4	4.9	11.5%
**Female in couple, has kids**	3.7	4.4	17.6%
**Single male, no kids**	2.9	3.2	11.3%
**Single male, has kids**	0.1	0.1	27.7%
**Male in couple, no kids**	5.2	5.5	6.2%
**Male in couple, has kids**	5.2	5.8	11.6%

Note: ‘All’ includes observations that have missing information on the various characteristics. Sample is individuals aged 25–64.

Source: Authors’ calculations using Labour Force Survey, 2007 and 2019.

TABLE A2. UK population by various characteristics, 2007 and 2019

	Percentage of population, 2007	Percentage of population, 2019	Difference (ppts)
**Female**	50.6%	50.5%	–0.1
**Male**	49.4%	49.5%	0.1
**Aged 25–34**	24.6%	26.0%	1.4
**Aged 35–44**	28.5%	24.3%	–4.2
**Aged 45–54**	24.6%	26.1%	1.5
**Aged 55–64**	22.3%	23.6%	1.3
**Degree**	23.2%	37.0%	13.8
**No degree**	76.8%	63.0%	–13.8
**White**	90.1%	86.4%	–3.7
**Non-white**	9.9%	13.5%	3.7
**Immigrant**	10.5%	15.9%	5.4
**Non-immigrant**	89.5%	84.1%	–5.4
**Single female, no kids**	9.8%	10.7%	0.8
**Single female, has kids**	5.2%	4.7%	–0.4
**Female in couple, no kids**	19.5%	18.6%	–0.9
**Female in couple, has kids**	16.1%	16.5%	0.4
**Single male, no kids**	12.2%	12.6%	0.4
**Single male, has kids**	0.5%	0.4%	–0.1
**Male in couple, no kids**	19.3%	18.5%	–0.8
**Male in couple, has kids**	17.4%	17.9%	0.5

Note: In order to calculate the percentage of people with(out) a degree, we drop those for whom we do not observe education. The whole sample includes observations that have missing information on the various characteristics. Sample is individuals aged 25–64.

Source: Authors' calculations using Labour Force Survey, 2007 and 2019.

TABLE A3. Change in employment rates amongst couples, 2007 to 2019

	Both are in work	One member of the couple is in work, one out	Both are out of work
2007	68%	24%	8%
2019	74%	21%	5%

Note: The sample comprises individuals aged 25–64 in a couple for whom we have information on their partner.

Source: Authors' calculations using Labour Force Survey, 2007 and 2019.

FIGURE A1. Change in part-time employment by various characteristics, 2007 to 2019

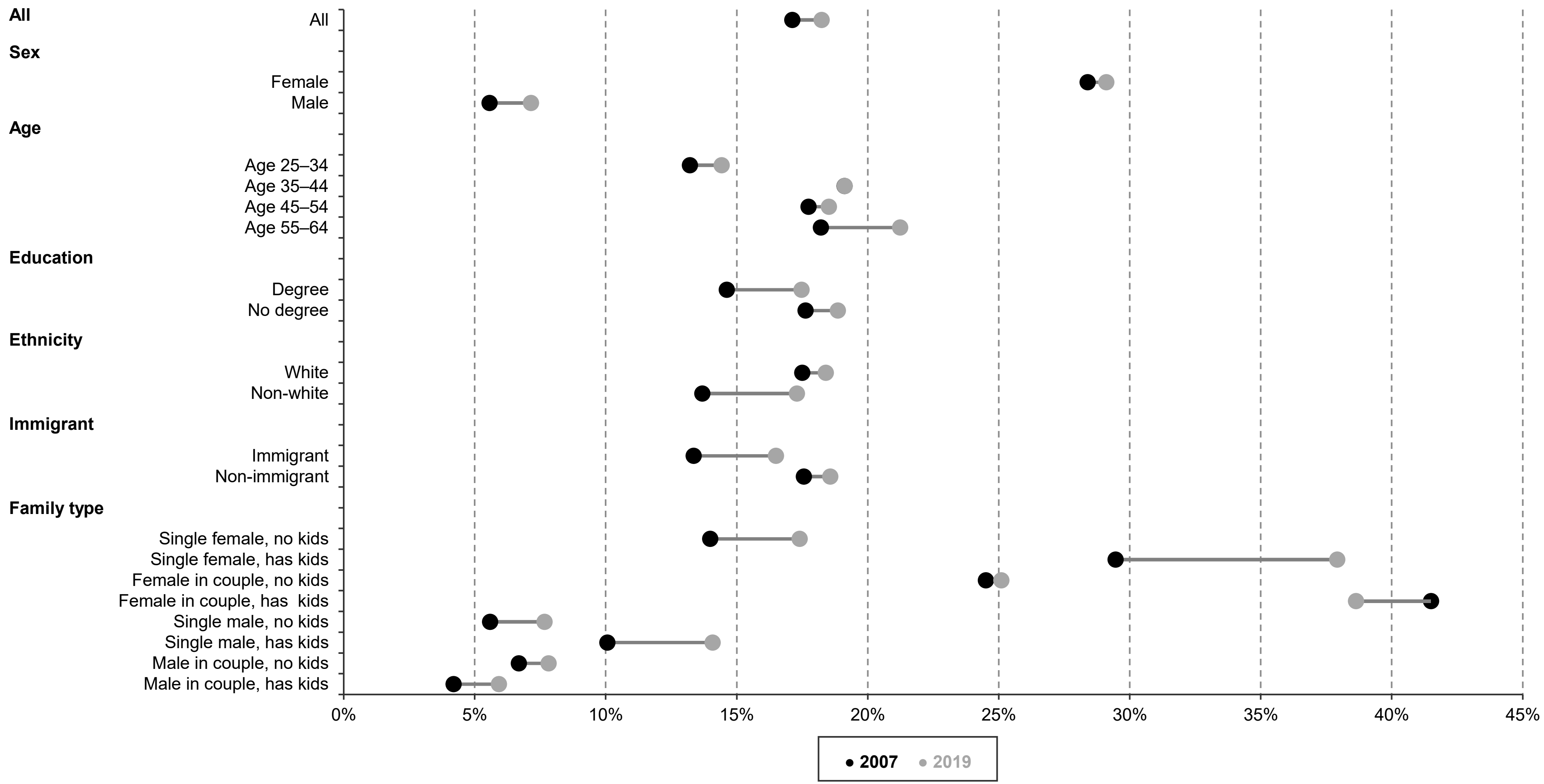

Note: 'Immigrants' are defined as those who first lived in the UK aged 16 or older. Sample is individuals aged 25–64.

Source: Authors' calculations using Labour Force Survey, 2007 and 2019.

FIGURE A2. Change in full-time employment by various characteristics, 2007 to 2019

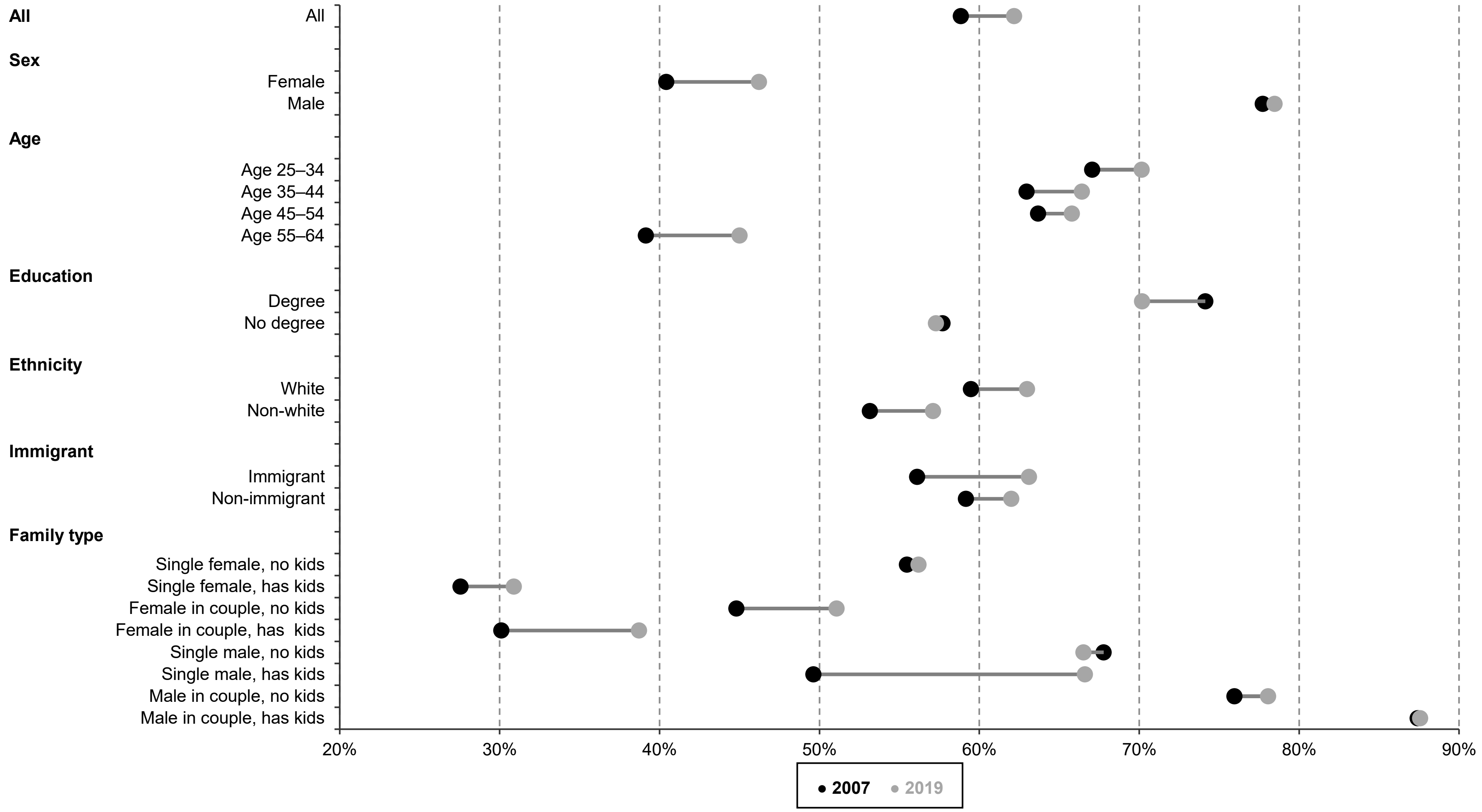

Note: 'Immigrants' are defined as those who first lived in the UK aged 16 or older. Sample is individuals aged 25–64.

Source: Authors' calculations using Labour Force Survey, 2007 and 2019.
